# Supplementary material for: Evaluation of the Predictive Ability, Environmental Regulation and Pharmacogenetics Utility of a BMI-Predisposing Genetic Risk Score during Childhood and Puberty
Source: J Clin Med. 2020 Jun 2;9(6):1705. doi: 10.3390/jcm9061705 (PMC7355743; doi:10.3390/jcm9061705)
Supplement: Supplementary file 1 [file jcm-09-01705-s001.pdf]

Supplementary Figure S1. Density distribution plots for the constructed pGRS according to the obesity status in study population 1. (A) Density is distribution plot for normal weight and children with obesity. (B) Density distribution plot with the inclusion of overweight individuals.

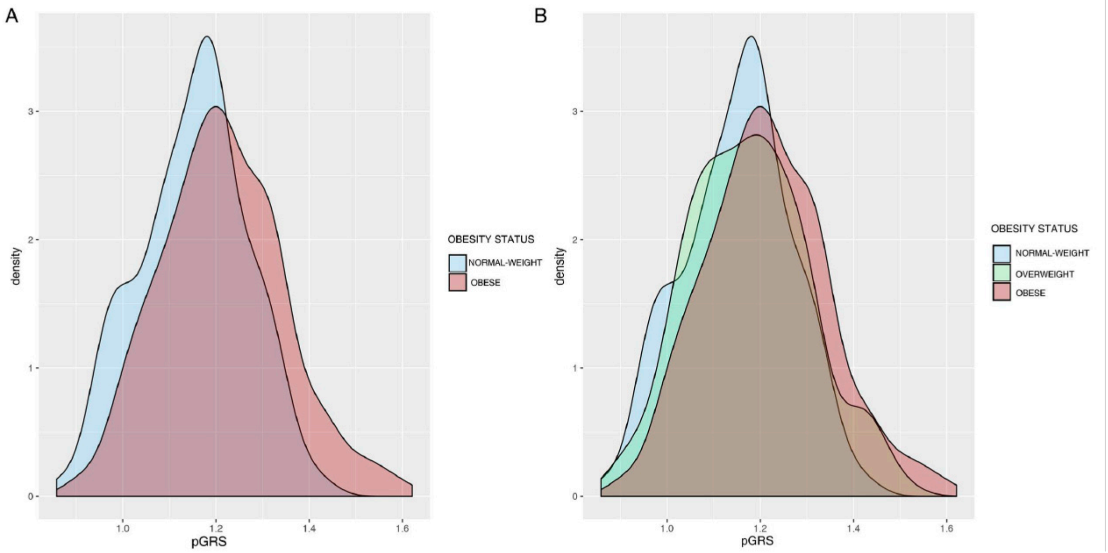

Supplementary Figure S2. Bar plot showing the number of normal weight, overweight and children with obesity according to each quartile of the pGRS in the study population 1.

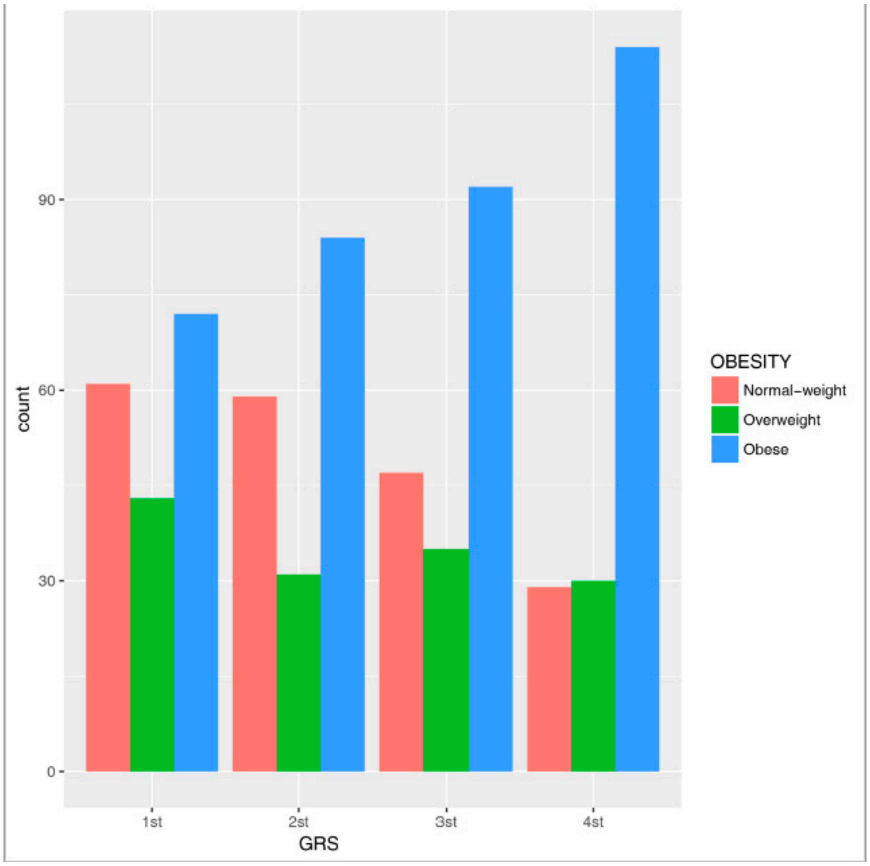

Supplementary Figure S3. Stepwise linear regression including all 44 tested SNPs in order to know which of them contribute the most to the pGRS-BMI association. Analysis performed in the study population 1.

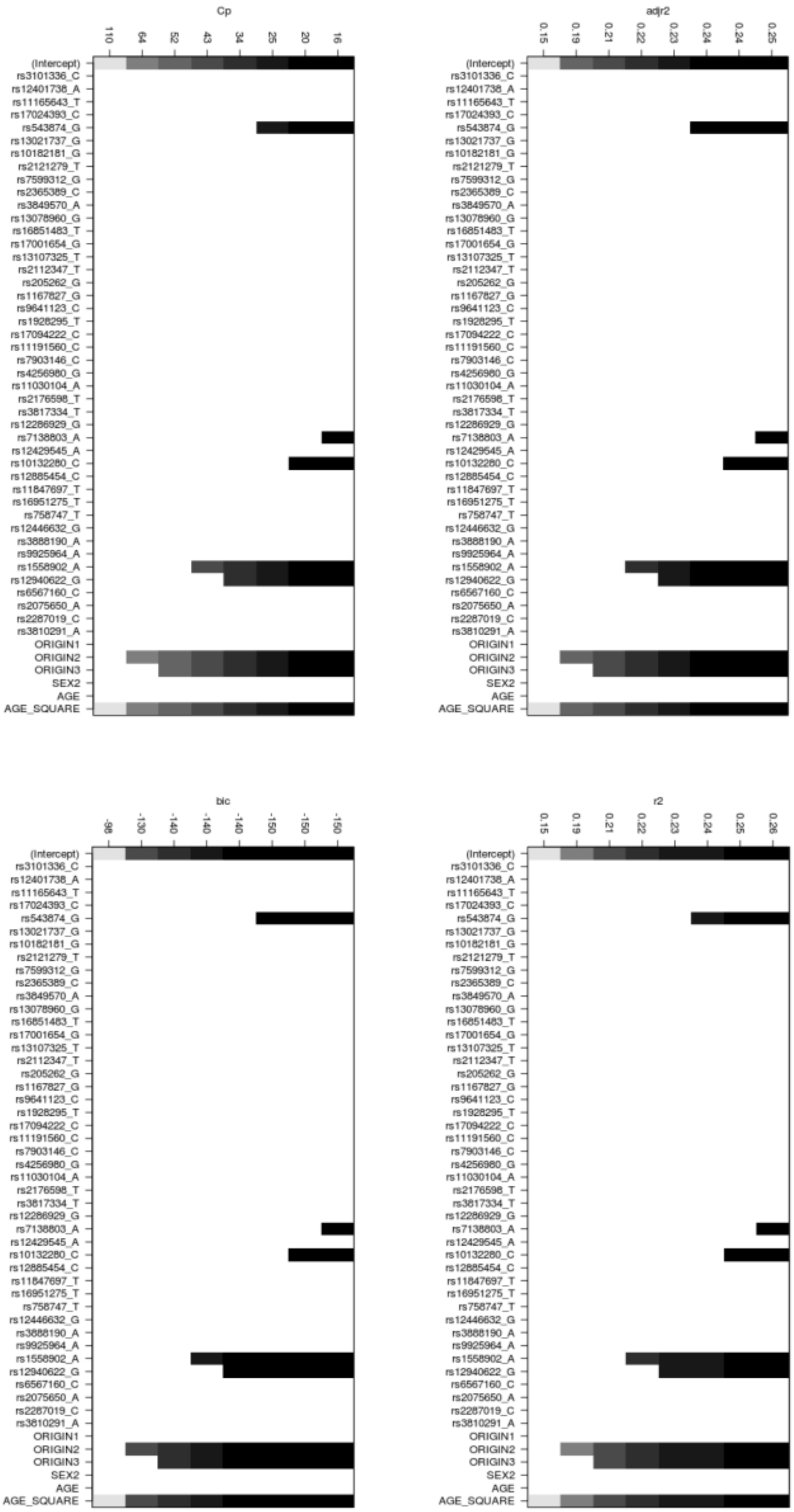

Supplementary Table S1. General characteristics, anthropometry, biochemical parameters, adipokines and cardiovascular/pro-inflammatory biomarkers in the cross-sectional cohort of 574 children (study population 1).

Phenotype	Normal weight	Overweight	Obesity	P-Value	FDR
Sex (Boys/Girls)	107/86 [a]	56/79 [b]	118/128 [ab]	0.04	0.04
BMI Z-Score	-0.27 (0.51) [c]	1.29 (0.48) [b]	3.46 (1.45) [a]	1.56E-141	3.90E-140
WC (cm)	59.44 (7.58) [c]	73 (11.28) [b]	86.36 (12.75) [a]	2.51E-84	3.14E-83
SBP (mmHg)	98 [48-120] [a]	105 [63-150] [b]	113 [70-155] [c]	9.00E-26	4.50E-25
DBP (mmHg)	59.77 (8.94) [c]	63.24 (10.83) [b]	67.94 (11.7) [a]	8.77E-14	1.83E-13
Fasting glucose (mg/dL)	84 [69-105] [ab]	85 [39-111] [a]	83 [59-109] [b]	1.34E-03	0.002
Fasting insulin (mU/L)	6.66 (4.21) [c]	9.58 (6.89) [b]	13.5 (9.49) [a]	2.95E-20	1.23E-19
QUICKI	0.38 [0.31-0.5] [a]	0.35 [0.28-0.45] [b]	0.34 [0.26-0.48] [c]	9.63E-20	3.44E-19
HOMA-IR index	1.41 (0.94) [c]	2.07 (1.6) [b]	2.84 (2.18) [a]	1.38E-17	4.31E-17
Total cholesterol (mg/dL)	169.04 (30.44) [a]	170.66 (35.13) [a]	164.17 (26.18) [a]	0.08	0.08
Triglycerides (mg/dL)	54.06 (21.87) [c]	67.2 (32.52) [b]	75.48 (35.49) [a]	1.64E-13	3.15E-13
HDLc (mg/dL)	65.76 (14.84) [a]	56.23 (13.32) [b]	49.53 (12.34) [c]	1.30E-26	8.13E-26
LDLc (mg/dL)	91.82 (26.3) [a]	98.35 (31.21) [a]	96.63 (23.11) [a]	0.08	0.08
Apo A1 (mg/dL)	155 [33-265] [a]	140 [31-209] [b]	131 [32-195] [c]	1.21E-16	3.36E-16
Adiponectin (mg/dL)	21.5 (11.45) [a]	20.16 (11.5) [a]	16.7 (9.78) [b]	1.03E-05	1.51E-05
Leptin (μg/L)	4.01 (4.27) [c]	10.97 (6.73) [b]	24.53 (15.64) [a]	3.74E-61	3.12E-60
Adiponectin-Leptin Ratio	0.25 (0.34) [b]	0.78 (0.69) [ab]	3.15 (14.06) [a]	1.81E-15	4.11E-15
Resistin (μg/L)	10.59 [0.26-102.03] [a]	12.03 [3.11-85.32] [a]	12.07 [0.41-71.79] [a]	0.56	0.56
MPO (μg/L)	10.56 [1.6-287.18] [a]	12.88 [0.01-222.13] [a]	20.44 [1.17-536.13] [b]	3.56E-06	5.56E-06
tPAI-1 (μg/L)	17.4 (12.74) [c]	22.48 (14.6) [b]	27.88 (17.61) [a]	2.47E-11	4.41E-11
TNF-α (ng/L)	2.4 [0.26-8.27] [a]	2.25 [0.39-12.68] [a]	3.42 [0.07-14.89] [b]	3.27E-08	5.45E-08
hsCRP(mg/L)	0.73 (1.33) [b]	1.52 (2.2) [b]	3.71 (4.78) [a]	1.10E-15	2.75E-15
MCP1 (ng/L)	86.91 [22.09-349.29] [ab]	84.86 [7-287.2] [a]	98.43 [13.37-358.2] [b]	0.02	0.02
IL-8 (ng/L)	1.71 (1.64) [b]	1.91 (1.96) [ab]	2.33 (2.34) [a]	5.07E-03	0.006
sICAM1 (mg/L)	0.13 [0.01-0.42] [a]	0.13 [0.04-0.5] [a]	0.14 [0.02-0.56] [a]	0.02	0.02

Data are expressed as mean (standard deviation) or median [min-max] if not normally distributed. One-way anova, Kruskal-Wallis and the Welch test were employed to assess group differences. Distributions within the same row with unlike superscript letters were significantly different (P-value<0.05) according to the post-hoc pairwise-t-tests, pairwise Mann–Whitney U-tests and Dunn tests. Childhood obesity was defined according to Cole et al. (2000). Abbreviations: Apo, apolipoprotein; BMI, body mass index; DBP, diastolic blood pressure; HDLc, high-density lipoproteins-cholesterol; HOMA-IR, homeostasis model assessment for insulin resistance; hsCRP, high-sensitivity C reactive protein; IL, interleukin; IR, insulin resistance; LDLc, low-density lipoproteins-cholesterol; MCP1, monocyte chemoattractant protein 1; MPO, myeloperoxidase; PAI-1, plasminogen activator inhibitor-1; QUICKI, quantitative insulin sensitivity check index; SBP, systolic blood pressure; sICAM, soluble intercellular cell adhesion molecule-1; TNF-α, tumour necrosis factor alpha; WC, waist circumference.

Supplementary Table S2. Descriptive statistics for the longitudinal study population 2.

	NW non-IR no change GROUP 1		OB/OW non-IR no change GROUP 3		OB/OW IR to non-IR GROUP 4		OB/OW non-IR to IR GROUP 5		OB/OW IR no change GROUP 6		FDR §
	T_0	Δ ($T_1 - T_0$)	T_0	Δ ($T_1 - T_0$)	T_0	Δ ($T_1 - T_0$)	T_0	Δ ($T_1 - T_0$)	T_0	Δ ($T_1 - T_0$)	
**N**	23		19		10		13		11		-
**Sex (♂/♀)**	(11/12)		(13/6)		(5/5)		(4/9)		(5/6)		0.71
**Age (y)**	8.5 [5,12.1]	6.77 (2.64)**** ^{b}	8.3 [4.6,12.1]	6.57 (2.43)**** ^{b}	8.1 [5,11.9]	6.3 (2.63)**** ^{ab}	7.8 [4,10.6]	6.12 (2.19)**** ^{b}	8.4 [7.3,13.3]	4.47 (1.31)**** ^{a}	9.50E-05
**WC (cm)**	57.75 (4.6) ^{b}	9.77 (7.02)**** ^{b}	79.16 (12.25) ^{a}	10.13 (12.4)** ^{ab}	80.78 (8.43) ^{a}	14.23 (12.17)** ^{ab}	75.7 (9.24) ^{a}	15.75 (8.51)**** ^{a}	81.66 (12.54) ^{a}	15.23 (9.92)*** ^{ab}	0.03
**HC (cm)**	67.34 (7.13) ^{c}	23.2 [8.7,31.67]	82.1 (8.24) ^{ab}	17.5 [2.5,51.55]	86.1 (6.39) ^{a}	19.67 [4,28.23]	76.8 (6.07) ^{b}	17.5 [10.57,39.03]	88.4 (7.09) ^{a}	14 [6.05,31.17]	0.75
**BMI Z-Score**	-0.43 (0.53) ^{b}	0.01 (0.06) ^{a}	2.74 (1.67) ^{a}	-0.09 (0.25) ^{a}	3.11 (1.97) ^{a}	-0.1 (0.2) ^{a}	2.41 (1.13) ^{a}	0.04 (0.15) ^{a}	2.84 (1.38) ^{a}	-0.01 (0.13) ^{a}	0.04
**DBP (mm Hg)**	62 [50,76]	7.5 [-12,55]**	62 [46,78]	6.75 [-12,27]**	71 [50,79]	5 [-18,31.5]	62 [45,80]	9 [-26,24]	65 [50,100]	12 [-5.5,22.5]*	0.71
**SBP (mm Hg)**	99 [79,120]	2.93 (16.21) ^{a}	105.5 [70,130]	11.5 (16)** ^{b}	110 [90,124]	5.61 (14.93) ^{ab}	105 [90,117]	10.19 (7.01)*** ^{b}	106 [72,144]	16.73 (21.68)* ^{b}	0.04
**Glucose (mg/dL)**	86 [78,98] ^{a}	0 [-16,25] ^{ab}	81 [73,96] ^{a}	3 [-20,15] ^{abc}	85 [78,95] ^{a}	-3 [-11.4] ^{a}	81 [68,92] ^{a}	7 [-11,24]* ^{c}	91 [79,109] ^{a}	3 [-25,19] ^{bc}	1.76E-03
**Insulin (mU/L)**	4.49 [1.99,15.22] ^{a}	3.44 (4.21)*** ^{c}	6.8 [2.2,17.13]** ^{ab}	2.95 (4.31) ^{c}	14.1 [12.8,32.67] ^{c}	-4.55 (9.81) ^{b}	8.8 [2.8,11.8] ^{b}	18.81 (8.69)**** ^{d}	14.93 [11.97,27.5] ^{c}	8.74 (3.44)**** ^{a}	1.79E-12
**QUICKI**	0.39 (0.04) ^{a}	-0.04 (0.04) ^{a}	0.37 (0.03) ^{ab}	-0.03 (0.04) ^{a}	0.32 (0.02) ^{c}	0.01 (0.03) ^{b}	0.36 (0.03) ^{b}	-0.06 (0.03) ^{c}	0.31 (0.01) ^{c}	-0.02 (0.01) ^{a}	5.03E-11
**HOMA-IR**	0.9 [0.39,3.53] ^{a}	0.77 (0.98) ^{c}	1.34 [0.4,3.63] ^{a}	0.62 (0.94)^{c}	3.1 [2.53,7.66]^{b}	-1.09 (2.19)^{b}	1.78 [0.47,2.36]^{a}	4.27 (2.23) ^{d}	4.01 [2.52,6.06] ^{b}	2.24 (1.12) ^{a}	1.67E-11
**Total Cholesterol (mg/dL)**	172 [129,229]	-5 [-40,41] ^{a}	173 [130,298]	-28 [-101,8]*** ^{b}	171 [141,221]	-22.5 [-59,14]* ^{ab}	149 [102,205]	1 [-30,33] ^{a}	180 [114,203]	-3 [-57,29] ^{a}	4.13E-04
**TAG (mg/dL)**	48.35 (13.45) ^{b}	15 [-31,146] ^{ab}	56.58 (11.98) ^{ab}	8 [-11,67] ^{ab}	81.4 (35.96) ^{a}	-2.5 [-71,35] ^{a}	60.31 (41.05) ^{ab}	24 [-61,77] ^{b}	76.45 (38.46) ^{a}	18 [-23,72] ^{ab}	8.81E-03
**HDLc (mg/dL)**	67.1 (15.11) ^{a}	-2 [-37,9] ^{a}	55.95 (12.18) ^{ab}	-10 [-25,7]*** ^{b}	54.9 (8.56) ^{ab}	-7.5 [-28, -2]** ^{ab}	46 (12.38) ^{b}	-3 [-20,8] ^{ab}	53.64 (13.84) ^{ab}	-4 [-17,9] ^{ab}	5.07E-03
**LDLc (mg/dL)**	97 [45,140]	-4.8 [-34.4,31] ^{ab}	107 [71,224]	-14.2 [-81,4.4]** ^{a}	99 [66,155]	-19 [-35,17.6]* ^{ab}	90 [52,139]	-3 [-31,28] ^{b}	103 [52,149]	3 [-44.6,27.6] ^{ab}	0.02

Baseline data are expressed as mean (standard deviation) or median [min-max] if not normally distributed. For Δ ($T_1 - T_0$) changes, data are expressed as mean change accompanied by [CI low, CI high]. Distributions within the same row with unlike superscript letters were significantly different (P-value < 0.05). * for P ≤ 0.05 in within-group changes (Δ) from start. ** for P-value ≤ 0.01 in for within-group changes (Δ) from start. *** for P-value ≤ 0.0001 in for within-group changes (Δ) from start. Within-group changes from baseline (T_0) to puberty (T_1) were assessed by means of a paired design in all continuous variables; employing either a paired

t-test or a Wilcoxon signed-rank test. Between-group differences were assessed by the one-way ANOVA, Kruskal-Wallis or Welch tests to the computed delta values (T_1–T_0) for each continuous measurement. § refers to FDR for between group Delta comparisons. Abbreviations: BMI, body mass index; CI, confidence interval; DBP, diastolic blood pressure; FDR, false discovery rate; HC, hip circumference, HDLc, high-density lipoproteins-cholesterol; HOMA-IR, homeostasis model assessment for insulin resistance; LDLc, low-density lipoproteins-cholesterol; QUICKI, quantitative insulin sensitivity check index; SBP, systolic blood pressure; TAG, triglycerides; WC, waist circumference.

Supplementary Table S3. Clinical characteristics of the study population 3 at baseline and post-treatment stages.

	Baseline			Post-treatment		
	Placebo N = 59	Metformin N = 65	P-value [1]	Placebo N = 59	Metformin N = 65	P-value [2]
**General Characteristics**						
Center (RS/VN/LB/US)	14/18/13/14	18/17/16/14	0.91			
Sex (Boys/Girls)	29/30	32/33	0.57			
Pubertal stage (Prepubertal/Pubertal)	28/31	35/30	0.30			
Age (y)	11.72 (2.10)	11.21 (2.17)	0.19			
Adherence (%)				85.78 (19.03)	92.38 (12.58)	**0.036**
**Outcomes**						
BMI Z score	3.51 (0.98)	3.32 (1.26)	0.16	3.11 (0.91)	2.7 (1.33)	**0.047**
Waist circumference (cm)	96.1 (9.37)	92.63 (10.97)	0.08	95.88 (9.82)	90.77 (11.09)	0.80
Fat mass (%)	38.12 (4.83)	36.61 (6.74)	0.16	36.97 (5.76)	36.65 (5.62)	0.84
DBP (mmHg)	68.49 (10.25)	68.82 (8.69)	0.85	68.62 (10.31)	67.38 (9.22)	0.29
SBP (mmHg)	118.29 (12.97)	115.13 (11.59)	0.17	115.09 (12.9)	112.33 (13.09)	0.77
Fasting glucose (mg/dL)	86.58 (7.24)	85.83 (6.87)	0.56	84.02 (8.89)	84.58 (9.4)	0.74
Fasting insulin (μU/mL)	17.42 (9.56)	16.15 (9.85)	0.38	17.27 (10.1)	16.39 (9.81)	0.82
HOMA-IR index	3.74 (2.2)	3.44 (2.15)	0.42	3.64 (2.28)	3.48 (2.22)	0.85
QUICKI	0.32 (0.04)	0.33 (0.03)	0.33	0.33 (0.03)	0.33 (0.03)	0.86
Total cholesterol (mg/dL)	162.86 (26.62)	158.44 (30.8)	0.41	157.32 (25.11)	157.33 (28.18)	0.17
Triglycerides (mg/dL)	76.6 (32.47)	75.66 (43.76)	0.49	71.57 (25.75)	76.26 (46.23)	0.87
HDLc (mg/dL)	46.79 (10.78)	45.73 (9.49)	0.57	48.02 (11.41)	49.61 (11.68)	**0.015**
LDLc (mg/dL)	99.33 (25.14)	96.41 (26.67)	0.54	93.52 (22.79)	91.44 (25.71)	0.54
VLDLc (mg/dL)	15.23 (7.64)	14.9 (9.14)	0.43	13.41 (4.37)	13.7 (8.25)	0.19
Apo A1 (mg/dL)	129.32 (19.83)	129.81 (21.2)	0.91	135.9 (19.85)	138.64 (22.68)	0.33
Apo B (mg/dL)	72.41 (15.31)	70.09 (14.82)	0.55	72 (10.42)	74.48 (19.29)	0.18
Adiponectin (mg/dL)	9.74 (6.67)	10.99 (5.64)	0.09	9.81 (7.14)	13.38 (6.73)	0.19
Leptin (μg/L)	16.95 (6.09)	15.08 (8.76)	0.18	14.16 (5.72)	14.26 (10.31)	0.32
ALR*	0.66 (0.5)	1.06 (1.03)	**0.005**	0.82 (0.69)	2.02 (2.83)	**0.009**
Resistin (μg/L)	12.72 (6.19)	13 (6.20)	0.81	13.39 (7.42)	13.39 (7.42)	0.30
MPO (μg/L)	394.19 (914.4)	199.5 (307.8)	0.88	111.83 (185.9)	182.05 (690.6)	0.06
tPAI-1 (μg/L)	28.85 (16.14)	26.29 (14.89)	0.46	23.55 (13.43)	21.21 (10.94)	0.45
TNF-α (ng/L)	8.38 (3.16)	8.35 (3.62)	0.95	6.49 (2.89)	6.11 (3.21)	0.77
IFN-γ (ng/L)	11.57 (13.89)	18.48 (31.84)	0.22	14.95 (36.76)	13.31 (32.34)	0.18
CRP (mg/L)	0.38 (0.6)	0.3 (0.31)	0.48	0.38 (0.67)	0.51 (1.06)	0.22
MCP-1 (ng/L)	194.19 (60.48)	190.35 (71.6)	0.66	173.93 (71.77)	167.61 (53.94)	0.62
IL-8 (ng/L)	3.86 (4.10)	3.39 (2.84)	0.93	2.95 (3.79)	2.26 (3.10)	0.62
sICAM-1 (μg/L)	108.03 (45.79)	110.82 (39.95)	0.47	87.14 (36.39)	94.21 (43.08)	0.82
sVCAM-1 (μg/L)	737.86 (252.24)	764.34 (215.23)	0.54	675.93 (188.64)	715.27 (212.63)	0.91

Data are expressed as mean (standard deviation) for continuous variables or *n* for categorical variables. [1] Differences in means between experimental groups at baseline were analyzed using the Student´s t-test or the U Mann Whitney test for quantitative variables, or the χ^2 test for categorical variables. [2] General linear model for repeated measures (MLG-MR) (with fixed effects: sex, pubertal stage, center, adherence (variables that did not influence the analysis were removed from the model to avoid over-adjustments), and time x treatment interaction; post hoc Bonferroni tests applied) were performed to analyze time differences between experimental groups (baseline vs. post-treatment) or ANCOVA (marked as *) when differences at baseline are observed between experimental groups. Abbreviations: ALR, adiponectin leptin ratio; Apo A1, apolipoprotein A1; Apo B, apolipoprotein B; BMI, body mass index; CRP, C reactive protein; DBP, diastolic blood pressure; IL-8, interleukin-8; HDLc, high-density lipoproteins-cholesterol; HOMA-IR, homeostasis model assessment for insulin resistance; INF-γ, interferon-γ; LB, Hospital Clínico Universitario Lozano-Blesa; LDLc, low-density lipoproteins-cholesterol; MCP-1, monocyte chemoattractant protein-1; MPO, myeloperoxidase; QUICKI, quantitative insulin sensitivity check index; RS, Hospital Universitario Reina Sofía; SBP,

systolic blood pressure; sICAM-1, soluble intercellular adhesion molecule-1, sVCAM-1, soluble vascular adhesion molecule-1; TNF-α, tumor necrosis factor-α; tPAI-1, total plasminogen activator inhibitor-1; US, Hospital Universitario de Santiago de Compostela; VLDLc, very low-density lipoproteins-cholesterol; VN, Hospital Universitario Virgen de las Nieves.

Supplementary Table S4. Hardy-Weinberg Equilibrium test for all analysed genetic variants in the original study population 1 [27].

CHR	SNP	TEST	A1	A2	GENO	O(HET)	E(HET)	P-VALUE	FDR
1	**rs11583200**	ALL	T	C	324/158/407	0.18	0.50	1.44E-84	2.42E-82
1	**rs11583200**	AFF	T	C	238/104/295	0.16	0.50	2.21E-66	1.86E-64
1	**rs11583200**	UNAFF	T	C	85/54/111	0.22	0.49	8.92E-18	5.00E-16
1	**rs3101336**	ALL	T	C	95/400/414	0.44	0.44	0.93	1.00
1	**rs3101336**	AFF	T	C	71/278/302	0.43	0.44	0.59	0.95
1	**rs3101336**	UNAFF	T	C	24/121/111	0.47	0.44	0.32	0.83
1	**rs12566985**	ALL	G	A	174/414/280	0.48	0.49	0.37	0.85
1	**rs12566985**	AFF	G	A	128/296/196	0.48	0.49	0.41	0.89
1	**rs12566985**	UNAFF	G	A	46/116/84	0.47	0.49	0.60	0.95
1	**rs12401738**	ALL	A	G	74/356/483	0.39	0.40	0.45	0.89
1	**rs12401738**	AFF	A	G	58/260/337	0.40	0.41	0.44	0.89
1	**rs12401738**	UNAFF	A	G	16/96/144	0.38	0.38	1	1.00
1	**rs11165643**	ALL	C	T	176/435/306	0.47	0.49	0.34	0.83
1	**rs11165643**	AFF	C	T	127/322/208	0.49	0.49	0.93	1.00
1	**rs11165643**	UNAFF	C	T	48/112/98	0.43	0.48	0.12	0.63
1	**rs17024393**	ALL	C	T	0/39/875	0.04	0.04	1	1.00
1	**rs17024393**	AFF	C	T	0/29/627	0.04	0.04	1	1.00
1	**rs17024393**	UNAFF	C	T	0/10/246	0.04	0.04	1	1.00
1	**rs543874**	ALL	G	A	32/248/623	0.27	0.29	0.24	0.75
1	**rs543874**	AFF	G	A	28/190/430	0.29	0.31	0.24	0.75
1	**rs543874**	UNAFF	G	A	4/57/192	0.23	0.22	1	1.00
1	**rs2820292**	ALL	A	C	219/428/238	0.48	0.50	0.34	0.83
1	**rs2820292**	AFF	A	C	157/298/178	0.47	0.50	0.15	0.66
1	**rs2820292**	UNAFF	A	C	62/129/59	0.52	0.50	0.70	0.98
2	**rs13021737**	ALL	A	G	25/238/628	0.27	0.27	0.62	0.95
2	**rs13021737**	AFF	A	G	16/156/462	0.25	0.25	0.52	0.92
2	**rs13021737**	UNAFF	A	G	9/80/166	0.31	0.31	1	1.00
2	**rs10182181**	ALL	G	A	218/456/236	0.50	0.50	1	1.00
2	**rs10182181**	AFF	G	A	161/322/167	0.50	0.50	0.81	1.00
2	**rs10182181**	UNAFF	G	A	57/134/67	0.52	0.50	0.61	0.95
2	**rs2121279**	ALL	T	C	8/177/732	0.19	0.19	0.59	0.95
2	**rs2121279**	AFF	T	C	5/137/515	0.21	0.20	0.24	0.75
2	**rs2121279**	UNAFF	T	C	3/40/215	0.16	0.16	0.43	0.89
2	**rs7599312**	ALL	A	G	53/302/558	0.33	0.35	0.15	0.66
2	**rs7599312**	AFF	A	G	36/217/400	0.33	0.34	0.36	0.84
2	**rs7599312**	UNAFF	A	G	17/84/157	0.33	0.35	0.21	0.75
3	**rs2365389**	ALL	T	C	128/438/346	0.48	0.47	0.62	0.95
3	**rs2365389**	AFF	T	C	79/320/254	0.49	0.46	0.17	0.71
3	**rs2365389**	UNAFF	T	C	49/117/91	0.46	0.49	0.30	0.83
3	**rs3849570**	ALL	A	C	100/415/381	0.46	0.45	0.45	0.89
3	**rs3849570**	AFF	A	C	81/290/271	0.45	0.46	0.79	1.00
3	**rs3849570**	UNAFF	A	C	19/123/110	0.49	0.43	0.06	0.39
3	**rs13078960**	ALL	G	T	53/315/535	0.35	0.36	0.45	0.89
3	**rs13078960**	AFF	G	T	46/225/375	0.35	0.37	0.13	0.64

3	**rs13078960**	UNAFF	G	T	7/89/159	0.35	0.32	0.24	0.75
3	**rs16851483**	ALL	T	G	4/130/778	0.14	0.14	0.81	1.00
3	**rs16851483**	AFF	T	G	3/102/548	0.16	0.15	0.60	0.95
3	**rs16851483**	UNAFF	T	G	1/28/228	0.11	0.11	0.59	0.95
3	**rs1516725**	ALL	T	C	7/149/667	0.18	0.18	0.84	1.00
3	**rs1516725**	AFF	T	C	4/101/482	0.17	0.17	0.80	1.00
3	**rs1516725**	UNAFF	T	C	3/47/184	0.20	0.20	1	1.00
4	**rs10938397**	ALL	G	A	193/422/294	0.46	0.49	0.07	0.44
4	**rs10938397**	AFF	G	A	131/312/208	0.48	0.49	0.47	0.89
4	**rs10938397**	UNAFF	G	A	62/109/85	0.43	0.50	0.02	0.22
4	**rs17001654**	ALL	G	C	43/303/556	0.34	0.34	0.84	1.00
4	**rs17001654**	AFF	G	C	34/219/392	0.34	0.35	0.64	0.96
4	**rs17001654**	UNAFF	G	C	9/84/162	0.33	0.32	0.84	1.00
4	**rs13107325**	ALL	T	C	11/154/748	0.17	0.17	0.33	0.83
4	**rs13107325**	AFF	T	C	6/116/531	0.18	0.18	1	1.00
4	**rs13107325**	UNAFF	T	C	5/38/215	0.15	0.17	0.05	0.39
5	**rs2112347**	ALL	G	T	89/392/419	0.44	0.43	0.87	1.00
5	**rs2112347**	AFF	G	T	59/265/320	0.41	0.42	0.70	0.98
5	**rs2112347**	UNAFF	G	T	29/126/99	0.50	0.46	0.27	0.78
6	**rs205262**	ALL	G	A	82/370/453	0.41	0.42	0.63	0.95
6	**rs205262**	AFF	G	A	61/261/327	0.40	0.42	0.39	0.89
6	**rs205262**	UNAFF	G	A	21/108/125	0.43	0.42	0.88	1.00
6	**rs2207139**	ALL	G	A	27/244/600	0.28	0.28	0.72	0.99
6	**rs2207139**	AFF	G	A	19/178/422	0.29	0.29	1	1.00
6	**rs2207139**	UNAFF	G	A	8/66/176	0.26	0.27	0.49	0.89
6	**rs9400239**	ALL	T	C	115/387/384	0.44	0.45	0.26	0.77
6	**rs9400239**	AFF	T	C	80/280/276	0.44	0.45	0.48	0.89
6	**rs9400239**	UNAFF	T	C	35/106/107	0.43	0.46	0.33	0.83
7	**rs1167827**	ALL	T	C	180/421/308	0.46	0.49	0.10	0.54
7	**rs1167827**	AFF	T	C	127/294/230	0.45	0.49	0.06	0.39
7	**rs1167827**	UNAFF	T	C	53/126/77	0.49	0.50	0.90	1.00
7	**rs9641123**	ALL	C	G	156/436/324	0.48	0.48	0.68	0.98
7	**rs9641123**	AFF	C	G	108/315/233	0.48	0.48	0.93	1.00
7	**rs9641123**	UNAFF	C	G	48/119/91	0.46	0.49	0.44	0.89
8	**rs17405819**	ALL	C	T	81/421/365	0.49	0.45	0.01	0.13
8	**rs17405819**	AFF	C	T	52/296/273	0.48	0.44	0.02	0.22
8	**rs17405819**	UNAFF	C	T	29/124/91	0.51	0.47	0.21	0.75
9	**rs10968576**	ALL	G	A	63/313/493	0.36	0.38	0.17	0.71
9	**rs10968576**	AFF	G	A	45/226/349	0.36	0.38	0.34	0.83
9	**rs10968576**	UNAFF	G	A	17/87/143	0.35	0.37	0.49	0.89
9	**rs1928295**	ALL	C	T	180/437/283	0.49	0.49	0.63	0.95
9	**rs1928295**	AFF	C	T	118/320/208	0.50	0.49	0.81	1.00
9	**rs1928295**	UNAFF	C	T	62/115/75	0.46	0.50	0.20	0.75
10	**rs17094222**	ALL	C	T	49/273/579	0.30	0.33	0.03	0.30
10	**rs17094222**	AFF	C	T	34/194/416	0.30	0.32	0.08	0.48
10	**rs17094222**	UNAFF	C	T	15/79/161	0.31	0.34	0.19	0.74
10	**rs11191560**	ALL	C	T	18/141/758	0.15	0.17	9.00E-4	0.02
10	**rs11191560**	AFF	C	T	14/105/538	0.16	0.18	4.19E-3	0.07
10	**rs11191560**	UNAFF	C	T	4/36/218	0.14	0.16	0.09	0.52
10	**rs7903146**	ALL	T	C	96/374/436	0.41	0.43	0.24	0.75
10	**rs7903146**	AFF	T	C	69/276/304	0.43	0.43	0.58	0.95
10	**rs7903146**	UNAFF	T	C	26/97/132	0.38	0.41	0.22	0.75
11	**rs4256980**	ALL	C	G	126/437/351	0.48	0.47	0.62	0.95
11	**rs4256980**	AFF	C	G	82/330/243	0.50	0.47	0.06	0.39
11	**rs4256980**	UNAFF	C	G	44/106/107	0.41	0.47	0.06	0.39
11	**rs11030104**	ALL	G	A	49/307/559	0.34	0.34	0.44	0.89

11	**rs11030104**	AFF	G	A	33/227/395	0.35	0.35	1	1.00
11	**rs11030104**	UNAFF	G	A	16/80/162	0.31	0.34	0.19	0.74
11	**rs2176598**	ALL	T	C	66/344/501	0.38	0.39	0.49	0.89
11	**rs2176598**	AFF	T	C	46/254/353	0.39	0.39	1	1.00
11	**rs2176598**	UNAFF	T	C	20/90/146	0.35	0.38	0.24	0.75
11	**rs3817334**	ALL	T	C	163/452/292	0.50	0.49	0.63	0.95
11	**rs3817334**	AFF	T	C	122/326/203	0.50	0.49	0.69	0.98
11	**rs3817334**	UNAFF	T	C	40/125/89	0.49	0.48	0.79	1.00
11	**rs12286929**	ALL	A	G	188/447/277	0.49	0.50	0.78	1.00
11	**rs12286929**	AFF	A	G	141/322/189	0.49	0.50	0.87	1.00
11	**rs12286929**	UNAFF	A	G	47/124/87	0.48	0.49	0.79	1.00
12	**rs7138803**	ALL	A	G	119/441/351	0.48	0.47	0.32	0.83
12	**rs7138803**	AFF	A	G	95/315/242	0.48	0.47	0.68	0.98
12	**rs7138803**	UNAFF	A	G	24/125/108	0.49	0.45	0.20	0.75
13	**rs12429545**	ALL	A	G	14/203/697	0.22	0.22	1	1.00
13	**rs12429545**	AFF	A	G	11/153/491	0.23	0.23	1	1.00
13	**rs12429545**	UNAFF	A	G	3/49/205	0.19	0.19	1	1.00
14	**rs10132280**	ALL	A	C	101/409/398	0.45	0.45	0.82	1.00
14	**rs10132280**	AFF	A	C	61/290/300	0.45	0.43	0.47	0.89
14	**rs10132280**	UNAFF	A	C	40/118/97	0.46	0.48	0.69	0.98
14	**rs12885454**	ALL	A	C	107/425/377	0.47	0.46	0.46	0.89
14	**rs12885454**	AFF	A	C	74/310/267	0.48	0.46	0.30	0.83
14	**rs12885454**	UNAFF	A	C	32/114/110	0.45	0.45	0.78	1.00
14	**rs11847697**	ALL	T	C	1/101/807	0.11	0.11	0.35	0.84
14	**rs11847697**	AFF	T	C	1/77/574	0.12	0.11	0.50	0.89
14	**rs11847697**	UNAFF	T	C	0/24/231	0.09	0.09	1	1.00
14	**rs7141420**	ALL	C	T	205/421/258	0.48	0.50	0.19	0.74
14	**rs7141420**	AFF	C	T	138/299/191	0.48	0.50	0.29	0.83
14	**rs7141420**	UNAFF	C	T	66/122/66	0.48	0.50	0.53	0.93
15	**rs16951275**	ALL	C	T	58/344/514	0.38	0.38	1	1.00
15	**rs16951275**	AFF	C	T	43/245/368	0.37	0.38	0.83	1.00
15	**rs16951275**	UNAFF	C	T	15/99/144	0.38	0.38	0.86	1.00
16	**rs758747**	ALL	T	C	93/365/449	0.40	0.42	0.15	0.66
16	**rs758747**	AFF	T	C	70/251/326	0.39	0.42	0.04	0.37
16	**rs758747**	UNAFF	T	C	22/114/122	0.44	0.42	0.56	0.95
16	**rs12446632**	ALL	A	G	16/200/693	0.22	0.22	0.65	0.97
16	**rs12446632**	AFF	A	G	10/152/490	0.23	0.23	0.73	1.00
16	**rs12446632**	UNAFF	A	G	6/48/201	0.19	0.21	0.13	0.64
16	**rs3888190**	ALL	A	C	117/404/392	0.44	0.45	0.42	0.89
16	**rs3888190**	AFF	A	C	87/296/271	0.45	0.46	0.67	0.98
16	**rs3888190**	UNAFF	A	C	30/108/119	0.42	0.44	0.47	0.89
16	**rs9925964**	ALL	G	A	146/426/326	0.47	0.48	0.72	0.99
16	**rs9925964**	AFF	G	A	116/302/225	0.47	0.49	0.41	0.89
16	**rs9925964**	UNAFF	G	A	30/123/100	0.49	0.46	0.49	0.89
16	**rs1558902**	ALL	A	T	208/451/251	0.50	0.50	0.84	1.00
16	**rs1558902**	AFF	A	T	160/326/167	0.50	0.50	1	1.00
16	**rs1558902**	UNAFF	A	T	48/123/84	0.48	0.49	0.79	1.00
17	**rs12940622**	ALL	G	A	167/385/338	0.43	0.48	2.73 E-3	0.05
17	**rs12940622**	AFF	G	A	130/279/232	0.44	0.49	7.43E-3	0.11
17	**rs12940622**	UNAFF	G	A	36/105/106	0.43	0.46	0.26	0.77
18	**rs1808579**	ALL	T	C	197/384/284	0.44	0.49	2.50E-3	0.05
18	**rs1808579**	AFF	T	C	137/283/200	0.46	0.49	0.05	0.39
18	**rs1808579**	UNAFF	T	C	60/100/83	0.41	0.50	9.46E-3	0.13
18	**rs6567160**	ALL	C	T	26/284/601	0.31	0.30	0.32	0.83
18	**rs6567160**	AFF	C	T	19/212/420	0.33	0.31	0.25	0.76
18	**rs6567160**	UNAFF	C	T	7/72/179	0.28	0.28	1	1.00

19	**rs29941**	ALL	G	A	38/364/435	0.43	0.39	3.47E-5	0.00
19	**rs29941**	AFF	G	A	29/262/309	0.44	0.39	4.69E-4	0.01
19	**rs29941**	UNAFF	G	A	9/101/126	0.43	0.38	0.05	0.39
19	**rs2075650**	ALL	G	A	13/123/776	0.13	0.15	5.00E-4	0.01
19	**rs2075650**	AFF	G	A	9/90/555	0.14	0.15	0.03	0.30
19	**rs2075650**	UNAFF	G	A	4/33/219	0.13	0.15	0.06	0.39
19	**rs2287019**	ALL	T	C	33/246/634	0.27	0.28	0.15	0.66
19	**rs2287019**	AFF	T	C	26/176/451	0.27	0.29	0.10	0.54
19	**rs2287019**	UNAFF	T	C	7/70/181	0.27	0.27	1	1.00
19	**rs3810291**	ALL	G	A	98/398/411	0.44	0.44	0.93	1.00
19	**rs3810291**	AFF	G	A	79/286/289	0.44	0.45	0.54	0.94
19	**rs3810291**	UNAFF	G	A	18/111/122	0.44	0.41	0.36	0.84

Each SNP has three entries showing results for either ALL individuals, AFF (overweight and children with obesity) or UNAFF (normal-BMI children only). Hardy Weinberg analysis was performed with the exact test described and implemented by Wigginton et al. (2005). Abbreviations; CHR, chromosome; SNP, single nucleotide polymorphism; A1, minor allele; A2, alternative allele; GENO, genotype counts; O(HET), observed heterozygosity; E(HET), expected heterozygosity; FDR, Hardy Weinberg false discovery rate.

Supplementary Table S5. List of 44 SNPs passing quality control filters and finally included in the Genetic Risk Score.

CHR	TYPE	LOCUS	SNP	REFERENCE ALLELE
chr1	intergenic	LOC101928241,PTBP2	rs11165643	T
chr1	intronic	DNAJB4	rs12401738	A
chr1	intronic	GNAT2	rs17024393	C
chr1	intergenic	NEGR1,LINC01360	rs3101336	C
chr1	intergenic	LOC101928778,SEC16B	rs543874	G
chr2	intergenic	ADCY3,DNAJC27	rs10182181	G
chr2	intergenic	FAM150B,TMEM18	rs13021737	G
chr2	intergenic	LRP1B,KYNU	rs2121279	T
chr2	intergenic	ERBB4,LOC102725079	rs7599312	G
chr3	intronic	CADM2	rs13078960	G
chr3	intronic	RASA2	rs16851483	T
chr3	intronic	FHIT	rs2365389	C
chr3	intronic	GBE1	rs3849570	A
chr4	exonic	SLC39A8	rs13107325	T
chr4	intronic	SCARB2	rs17001654	G
chr5	intergenic	POC5,SV2C	rs2112347	T
chr6	intronic	C6orf106	rs205262	G
chr7	UTR3	HIP1	rs1167827	G
chr7	intronic	CALCR	rs9641123	C
chr9	intergenic	ASTN2,LOC101928797	rs1928295	T
chr10	intronic	NT5C2	rs11191560	C
chr10	intergenic	HIF1AN,PAX2	rs17094222	C
chr10	intronic	TCF7L2	rs7903146	C
chr11	ncRNA_intronic	BDNF-AS	rs11030104	A
chr11	intergenic	NXPE2,CADM1	rs12286929	G
chr11	intronic	HSD17B12	rs2176598	T
chr11	intronic	MTCH2	rs3817334	T
chr11	intronic	TRIM66	rs4256980	G
chr12	intergenic	BCDIN3D,FAIM2	rs7138803	A
chr13	intergenic	LINC01065,LINC00558	rs12429545	A
chr14	intergenic	STXBP6,NOVA1	rs10132280	C

chr14	intergenic	PRKD1,G2E3	rs11847697	T
chr14	intergenic	LINC01551,PRKD1	rs12885454	C
chr15	intronic	MAP2K5	rs16951275	T
chr16	intergenic	GPRC5B,GPR139	rs12446632	G
chr16	intronic	FTO	rs1558902	A
chr16	upstream;downstream	ATP2A1;ATP2A1-AS1	rs3888190	A
chr16	UTR5	NLRC3	rs758747	T
chr16	intronic	KAT8	rs9925964	A
chr17	intronic	RPTOR	rs12940622	G
chr18	intergenic	PMAIP1,MC4R	rs6567160	C
chr19	intronic	TOMM40	rs2075650	A
chr19	intronic	QPCTL	rs2287019	C
chr19	UTR3	ZC3H4	rs3810291	A

Abbreviations; CHR, chromosome; SNP, single nucleotide polymorphism.

Supplementary Table S6. Lifestyle factors assessed in our study for the study population 1.

**Diagnosed hypertriglyceridemia in father or mother**
Do you usually eat in front of the TV?
Educational level of the father
Educational level of the mother
Father BMI
How long does it take to get to the school on walk?
How many days do you spend doing vigorous efforts like training activity?
how many days per week do you exercise in a sport club?
How many days per week do you spend doing home activities?
How many days per week do you spend doing physical activity in family?
How many days per week do you spend walking with vigorous efforts?
How many hours a day do you spend walking with vigorous efforts?
How many hours do you spend doing home activities?
How many hours do you spend doing homeworks outside of school hours?
How many hours do you spend doing physical activity in family?
How many hours do you spend exercising in a sport club?
How many hours do you usually sleep every day during the week?
How many hours do you usually sleep every day during the weekends?
How many hours each day do you spend doing vigorous efforts like training activity?
How many hours each day do you spend practising activities that do not require physical activity (e.g. reading
How many hours each day you spend walking quite a lot without vigorous efforts?
How many hours per week do you spend on physical education during school hours?
How many minutes per week do you spend exercising at a sport program?
How much time do you play videogames in a day during the week?
How much time do you play videogames in a day during weekend?
How much time during the week do you usually watch TV
How much time during the weekend do you spend watching TV and DVD?
How much time in per weekend do you usually use internet
How much time per day do you use internet during the week?
How much time per weekend do you usually use the smartphone?
How often do you eat candies while watching TV?
How often do you eat fruit while watching TV?
How often do you eat fruits while playing video games?
How often do you eat fruits while surfing internet?
How often do you eat nuts while watching TV?
How often do you eat salted potatoes while watching TV?
How often do you eat snacks while playing videogames?

How often do you eat snacks while surfing internet?
How often do you eat snacks while watching TV?
How often do you eat sweets while watching TV?
Mother BMI
Presence of diabetes in father or mother
Presence of heart stroke in father or mother
Presence of AH in father or mother
Presence of hypercholesterolemia in father or mother
Presence of obesity in the father or mother
Presence of vascular problems in father or mother

Abbreviations; AH, arterial hypertension; BMI, body mass index.

Supplementary Table S7. Single-SNP analyses on BMI Z-Score in the study population 1.

SNP	Beta	SE	CI.LO	CI.HI	T-Value	P-Value	FDR
**rs10132280_C_STXBP6,NOVA1**	0.75	0.29	0.18	1.33	2.57	**0.01**	0.15
**rs7138803_A_BCDIN3D,FAIM2**	0.72	0.29	0.14	1.29	2.43	**0.02**	0.15
**rs12401738_A_DNAJB4**	0.76	0.32	0.14	1.37	2.40	**0.02**	0.15
**rs543874_G_LOC101928778,SEC16B**	0.84	0.36	0.14	1.55	2.35	**0.02**	0.15
**rs2112347_T_POC5,SV2C**	0.68	0.30	0.10	1.26	2.28	**0.02**	0.15
**rs9925964_A_KAT8**	-0.65	0.29	-1.21	-0.09	-2.28	**0.02**	0.15
**rs3101336_C_NEGR1,LINC01360**	0.69	0.30	0.09	1.28	2.26	**0.02**	0.15
**rs1558902_A_FTO**	0.58	0.28	0.03	1.13	2.08	**0.04**	0.19
**rs12940622_G_RPTOR**	0.58	0.28	0.03	1.12	2.07	**0.04**	0.19
rs7599312_G_ERBB4,LOC102725079	0.61	0.34	-0.05	1.27	1.81	0.07	0.31
rs3849570_A_GBE1	0.51	0.30	-0.07	1.09	1.73	0.08	0.34
rs11847697_T_PRKD1,G2E3	0.97	0.63	-0.26	2.20	1.55	0.12	0.45
rs2365389_C_FHIT	0.44	0.29	-0.13	1.02	1.51	0.13	0.45
rs13021737_G_FAM150B,TMEM18	0.50	0.38	-0.24	1.24	1.33	0.18	0.57
rs17094222_C_HIF1AN,PAX2	-0.44	0.34	-1.11	0.22	-1.30	0.19	0.57
rs12286929_G_NXPE2,CADM1	-0.34	0.28	-0.89	0.21	-1.22	0.22	0.57
rs2075650_A_TOMM40	0.61	0.51	-0.38	1.60	1.21	0.23	0.57
rs12429545_A_LINC01065,LINC00558	0.50	0.42	-0.33	1.33	1.19	0.23	0.57
rs6567160_C_PMAIP1,MC4R	0.40	0.37	-0.34	1.13	1.06	0.29	0.65
rs2121279_T_LRP1B,KYNU	0.49	0.48	-0.44	1.43	1.03	0.30	0.65
rs9641123_C_CALCR	-0.29	0.29	-0.84	0.27	-1.00	0.32	0.65
rs3888190_A_ATP2A1;ATP2A1-AS1	0.29	0.29	-0.28	0.86	0.99	0.32	0.65
rs2287019_C_QPCTL	0.31	0.37	-0.42	1.04	0.84	0.40	0.71
rs205262_G_C6orf106	-0.25	0.30	-0.84	0.34	-0.83	0.41	0.71
rs13078960_G_CADM2	0.28	0.33	-0.37	0.92	0.83	0.41	0.71
rs16851483_T_RASA2	0.45	0.57	-0.67	1.57	0.79	0.43	0.73
rs12885454_C_LINC01551,PRKD1	-0.21	0.30	-0.80	0.38	-0.69	0.49	0.76
rs3810291_A_ZC3H4	-0.20	0.30	-0.79	0.38	-0.68	0.50	0.76
rs13107325_T_SLC39A8	0.31	0.46	-0.60	1.21	0.66	0.51	0.76
rs3817334_T_MTCH2	0.18	0.29	-0.38	0.74	0.62	0.54	0.76
rs1928295_T_ASTN2,LOC101928797	0.17	0.28	-0.38	0.73	0.61	0.54	0.76
rs758747_T_NLRC3	-0.18	0.30	-0.77	0.41	-0.59	0.55	0.76
rs4256980_G_TRIM66	-0.17	0.29	-0.74	0.40	-0.57	0.57	0.76
rs10182181_G_ADCY3,DNAJC27	0.14	0.28	-0.41	0.69	0.50	0.62	0.76
rs11165643_T_LOC101928241,PTBP2	0.14	0.28	-0.41	0.69	0.49	0.62	0.76
rs17001654_G_SCARB2	0.16	0.34	-0.50	0.83	0.48	0.63	0.76
rs7903146_C_TCF7L2	0.13	0.30	-0.45	0.72	0.44	0.66	0.76
rs12446632_G_GPRC5B,GPR139	0.18	0.42	-0.64	1.00	0.42	0.67	0.76
rs1167827_G_HIP1	0.12	0.28	-0.43	0.67	0.42	0.68	0.76

rs11191560_C_NT5C2	-0.13	0.44	-0.99	0.74	-0.29	0.77	0.85
rs17024393_C_GNAT2	-0.23	1.03	-2.25	1.78	-0.23	0.82	0.86
rs2176598_T_HSD17B12	-0.07	0.32	-0.69	0.56	-0.21	0.83	0.86
rs11030104_A_BDNF-AS	0.07	0.33	-0.59	0.72	0.20	0.84	0.86
rs16951275_T_MAP2K5	0.05	0.33	-0.58	0.69	0.16	0.87	0.87

SNPs in bold showed statistically significant associations with BMI Z-Score under a multiple linear regression adjusted for origin, sex and pubertal status. Each is SNP name is presented accompanied by the reference allele and the name of the nearest mapped loci. Abbreviations; SNP, single nucleotide polymorphism; SE, standard error; CI.LO, low confidence interval; CI.HI, high confidence interval; FDR, false discovery rate.

Supplementary Table S8. Association between the pGRS (quartilized) and the metabolic health status of children in the study population 1.

	**MUO**			**SBP**			**DBP**			**GLU**			**HOMA-IR**			TAG			HDLc		
	**B**	**SD**	**FDR**	**B**	**SD**	**FDR**	**B**	**SD**	**FDR**	**B**	**SD**	**FDR**	**B**	SD	FDR	B	SD	FDR	B	SD	FDR
GRS-Q2 vs GRS-Q1	-0.10	0.28	0.94	0.18	0.31	0.94	0.15	0.35	0.94	-0.03	0.58	0.99	0.18	0.35	0.94	-0.37	0.38	0.94	-1.21	0.62	0.53
GRS-Q3 vs GRS-Q1	-0.75	0.28	0.19	-0.32	0.34	0.94	-0.49	0.40	0.94	-0.33	0.61	0.94	-0.16	0.35	0.94	-0.49	0.39	0.94	-0.09	0.48	0.94
GRS-Q4 vs GRS-Q1	-0.25	0.29	0.94	0.16	0.31	0.94	0.19	0.34	0.94	-17.62	1441.82	0.99	0.06	0.34	0.94	-0.53	0.38	0.94	-0.11	0.45	0.94

For these analyses, the general metabolic health status as well as its six dichotomized components (high glucose, HOMA-IR, DBP, SBP or TAG values or low HDLc levels) were employed. Dichotomization of these metabolic outcomes was accomplished according to the criteria we have previously published [33]. Multiple logistic regression models were applied adjusted for BMI Z-Score, sex, age, origin and pubertal status of children. Abbreviations: B, beta; DBP, diastolic blood pressure; FDR, false discovery rate; GLU, glucose levels; HDLc, high-density lipoproteins-cholesterol; HOMA-IR, homeostasis model assessment for insulin resistance; SBP, systolic blood pressure; SD, standard deviation; MUO, metabolically unhealthy obese; TAG, triglycerides.
